# Computational Modelling of Imidazole Protection of Coordinated Gadolinium Tetraphenylporphyrine Against Molecular Oxygen Attack

**DOI:** 10.3390/molecules30214246

**Published:** 2025-10-31

**Authors:** Vladimir Pomogaev, Daniil Lukyanov, Elena Solovyeva

**Affiliations:** Institute of Chemistry, Saint-Petersburg State University, Universitetskaya nab. 7/9, 199034 Saint Petersburg, Russia; valienpo@yandex.ru (V.P.);

**Keywords:** porphyrin, gadolinium, imidazole, DFT, ESP, dioxygen

## Abstract

Promising photophysical properties and the enhanced sensitivity to molecular oxygen of porphyrins metalated with Gd(III) generate a need for their detailed description on an atomic level with the account of coordinated ligands, which also influence the properties. Herein, the complexation of tetraphenylporphyrin with gadolinium chloride in imidazole medium was analyzed using density functional theory in the framework of ωB97XD functional with hybrid diffused polarization-consistent basis sets. The complexes with different number of coordinated imidazole ligands (*k* = 0–2) were calculated to compare their structural parameters, electrostatic potential distribution, and interaction with molecular oxygen. Thermodynamic functions of complex formation were estimated for a set of possible reactions, including various side products (hydrogen chloride or imidazole hydrochloride) and different number of imidazole molecules involved. Weak interactions in the coordination sphere of chlorogadolinium tetraphenylporphyrin with attached imidazole ligands were also assessed. Performed analysis proved the presence of imidazole protection against the molecular oxygen attack.

## 1. Introduction

Metalloporphyrins are a class of complex compounds that play an important role in redox and photoinduced processes of nature and physiology. Photophysical properties of metalloporphyrins are determined by the metal center and substituents in the organic macrocycle and can be regulated by the variation in their combinations. Metalation of porphyrins with lanthanides allows achieving increased phosphorescence [1,2], which can be used in various applications of spectral analysis such as oxygen sensing [3,4,5,6] or dual-mode imaging [7,8]. The experimentally observed phosphorescence enhancement is particularly attractive in the case of porphyrin complexes with gadolinium, because it originates from not only the heavy atom effect but also due to the energy level of Gd3+, as well as mixing between the singlet and triplet states [9,10,11].

The structure of metalloporphyrins often includes non-coaxial ligands attached to the metal center, which may alter their optical and catalytic properties and modify the interaction with molecular oxygen on par with axial ligands. Imidazole is one of the most curious and frequently encountered non-coaxial ligands in the metal complexes of porphyrin. The demonstrative example is proximal histidine coordinated with iron via imidazole ring in heme, which regulates its position during the oxygenation–deoxygenation cycle. The influence of the coordinated imidazole ligands on catalytic activity has also been revealed for cobalt (II) [12,13], nickel (II) [14], and zinc(II) [15] metalloporphyrins. For porphyrin complexes with gadolinium, obtaining in molten imidazole by the Srivastava method [16], the inclusion of imidazole ligands in the coordination sphere has also been documented [17]. Moreover, on the example of gadolinium-coordinated hematoporphyrin monomethyl ether, it has been shown that imidazole contributes to the enhancement of phosphorescence by partially inhibiting energy transfer from the lowest triplet state of the complex to oxygen [18,19].

The detailed description of metalloporphyrins interaction with molecular oxygen is of huge fundamental and practical interest, but is insufficient today. The significance of having deep understanding of these interactions and the lack of experimental data on the atomic scale encourages numerous attempts at their computer modeling. Although such studies are distinguished by the use of high-level theory, they mostly deal with model metalloporphyrins without ligands. The coordination of non-coaxial ligands has been taken into account in only a few studies, providing valuable details about the stabilizing effect on the affinity for O_2_ [20,21,22,23]. For the metal complexes of porphyrin with gadolinium, there are no detailed calculations of the electronic properties, coordination with apical and non-coaxial ligands, and thermochemical parameters of complexation at all. However, their promising photophysical properties, with their enhanced sensitivity to dioxygen, motivated us to perform the quantum-chemical study of the model tetraphenylporphyrine complex with gadolinium chloride (ClGdTPP) to examine its coordination with imidazole and binding with molecular oxygen.

## 2. Results and Discussion

### 2.1. Molecular Structure and Spin Multiplicity

**Figure 1:**
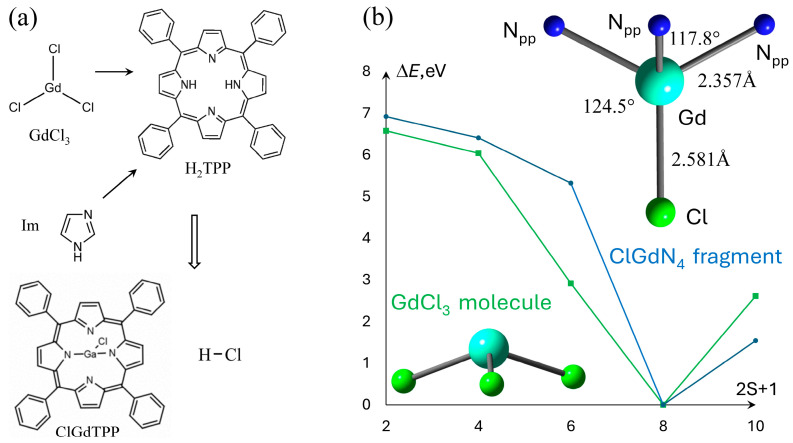
(**a**) 2D sketches of the reactants and products in ClGdTPP formation; (**b**) the dependence of ground state energy on ClGdTPP and GdCl_3_ multiplicity, Δ*E* = *E* − *E*_min_. The calculated coordination parameters of ClGdN_4_ fragment of ClGdTPP are indicated in the right part.

Gadolinium provides the highest spin multiplicity among lanthanides and all other metals for the Gd-containing compounds in the ground state. For instance, high-spin multiplicities in ferromagnetic ground state of supramolecular halide complexes based on the gadolinium chloride were recently noted [24]. This fact requires a total spin test for such systems. The total charge and Gd atom define the total spin of the whole neutral compound. ClGdTPP (Figure 1a) possess the highest total spin due to seven unpaired electrons on Gd 4*f* shell, which leads to two groups of molecular orbitals (MO) formed almost exclusively from *f*-atomic orbitals (AO), which are α singly occupied MOs and the corresponding energetically low-laying unoccupied β MOs. The neutral ClGdTPP complex with various multiplicities was optimized in the ground state using ωB97XD with def2VP (Figure 1b) and compared with the other data, especially with the much larger full-electron x2cVP basis sets to verify the most appropriate spin multiplicity for ClGdTPP minimum energy in comparison with the other results [1,24,25].

The Gd–Cl bond exclusively defines the energetically preferable spin multiplicity m = 8 of neutral ClGdTPP complex, as well as in its precursor GdCl_3_, which was calculated for comparison. The other multiplicities m = 4, 6, 10 provide the energy differences of 6.4, 5.3, 1.5 eV and 6.0, 2.9, 2.6 eV, respectively, for both compounds. The ClGdTPP and GdCl_3_ molecules optimized with m = 8 using a smaller def2VP basis set than the expensive large full-electron x2cVP provide almost the same structural parameters. The central Gd atom is placed out of the porphyrin plane and coordinated in a symmetrical manner with four near-equatorial nitrogen atoms of tetrapyrrole (N_pp_) and apical Cl atom placed vertically to the molecular plane. The distances |Gd–Cl| = 2.581 Å, |Gd–N_pp_| = 2.357 Å, and angles between these atoms ∠N_pp_GdCl = 117.8°, ∠N_pp_GdN_pp_ = 124.5° define a degree of non-planarity h_Gd_ = 1.098 Å, which is assumed as the Gd–N_pp_ length projection on the Gd–Cl axis. The proximity of the obtained structural parameters allowed us to calculate the large ClGdTPP complexes coordinated with several imidazole ligands and study a molecular oxygen attack on these structures in a computationally much faster and cheaper manner using the smaller def2VP.

### 2.2. ClGdTPP Formation and Coordination with Imidazole Ligands

Thermochemical studies reveal the most preferable reaction pathways from initial reagents to final products. The formation and coordination of metal complexes are based on calculations of the total heat and maximum work corresponding to the differences between standard enthalpies (Δ*H_T_* = Δ*H*°*_T_*) and standard Gibbs free energies (Δ*G_T_* = Δ*G*°*_T_*), which are (Δ*H*_535_ & Δ*G*_535_) at T = 535 K, respectively. Electrostatic potentials (ESP) minima and maxima define ligand coordination and oxygen attack. To calculate the thermodynamic functions of complexation of H_2_TPP with GdCl_3_ in boiling imidazole (Figure 1a), only real frequencies were obtained for all participants.

**Figure 2:**
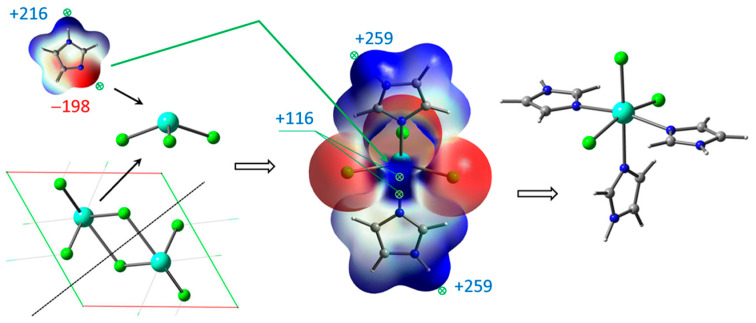
Unpacking GdCl_3_ crystal and coordination of Gd atom with imidazole ligands based on ESP mapping of max (+) and min (−) points for interaction.

The chosen setting of functional and basis sets should correctly calculate not only geometer parameters but also electronic MOs, electrostatic potentials, and thermochemical state functions for the compounds under study. Using the ωB97XD functional with smaller def2VP basis set, ClGdTPP complexes coordinated with one and two imidazole ligands were calculated. The expected final ClGdTPP·*k*Im complexes were obtained from GdCl_3_ coordinated with several Im moieties attacking H_2_TPP molecule. The initial processes in molten imidazole are GdCl_3_ crystal unpacking and formation of intermediate coordination complexes (Figure 2).

GdCl_3_ crystal unpacking is accompanied by cleavage of two Gd–Cl bonds, resulting in coordinatively unsaturated molecules, which readily form the complexes with several imidazole ligands. The complex GdCl_3_·2Im has the strong ESP maximum +115.8 kJ/mol near the metal center, providing a site for third imidazole ligand with ESP minimum −197.8 kJ/mol. This complex may turn out to be more suitable for the thermochemical interaction with H_2_TPP. The greater ESP maxima +258.6 kJ/mol and +260.0 kJ/mol near hydrogen atoms of H–N bonds do not play roles in complex formation. Thus, both GdCl_3_ complexes with 5- and 6-coordinated Gd atom were considered as intermediate species participating in final ClGdTPP formation. Several more energetically preferable reaction pathways are discussed and interpreted below based on ESP analysis and free energy differences Δ*G.*

**Figure 3:**
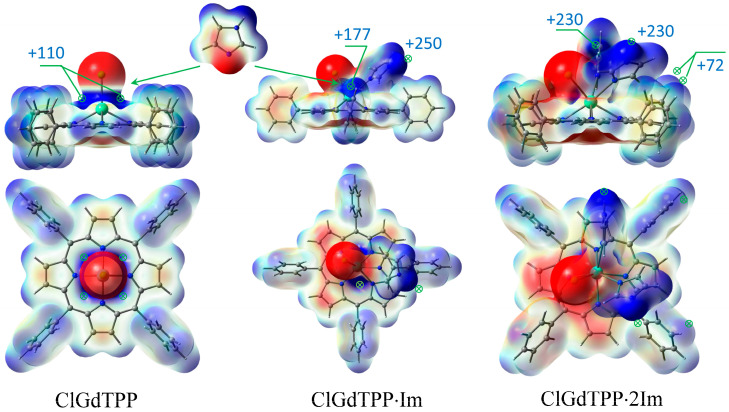
ESP maxima in kJ/mol with scale mapping from red to blue regions (−3 × 10^−2^ ÷ 3 × 10^−2^ a.u.) on isodensity = 0.004 ē/bohr^3^ at stepwise coordination of ClGdTPP with imidazole in top-facing and front-facing projections.

The coordination of ClGdTPP complex with Im ligands is highly expected in imidazole medium and it was also considered based on ESP analysis. Taking into account the apical Cl atom, the coordination of one or two imidazole molecules is possible in this case. Chlorine provides a vicinity of negative charge density but leaves quite an open positive space around Gd atom, with four points of the ESP maximum (+109.9 kJ/mol) for imidazole attachment to its negative nitrogen centers (Figure 2 and Figure 3). One imidazole molecule provides the attractive maximum +250.1 kJ/mol of its H–N bond and shields a part of the positive area, but there is still one accessible positive ESP point of +177.2 kJ/mol for another ligand attachment. Finally, a second imidazole molecule closes the site. The hindrance from the couple of imidazole ligands and the absence of a noticeable ESP maximum near Gd atom in ClGdTPP·2Im prevent further coordination. Thus, the highest coordination number of ClGdTPP is 7 in the case of two imidazole ligands, which complete the coordination surroundings. The maxima of +230.3 kJ/mol near imidazole ligands and 59.3 ÷ 72.1 kJ/mol close to hydrogen atoms of terminal benzine rings provide interaction only at these points, which is an additional protection by Gd atom against dioxygen attack.

**Figure 4:**
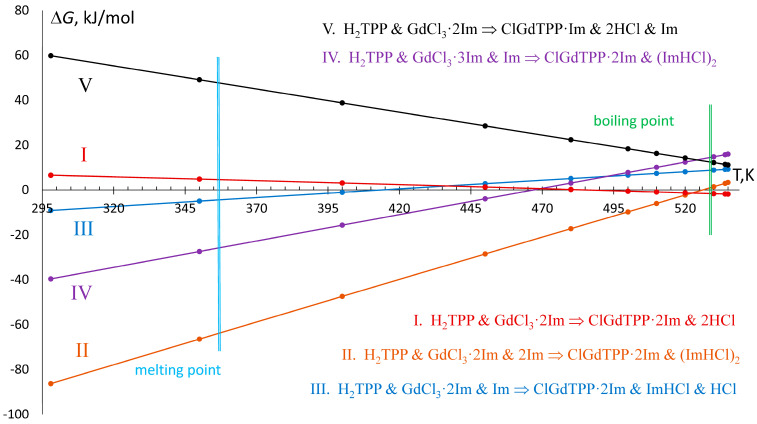
Temperature dependence of Gibbs free energy of the most favorable in synthetic conditions reactions, those equations are indicated in the right part.

Summing up the ESP analysis results, it can be concluded that the formation of 7-coordinated ClGdTPP·2Im complex from initial H_2_TPP and GdCl_3_ is energetically preferable. The most favorable reactions (Figure 4) at 535 K involve intermediate GdCl_3_·2Im, rather than GdCl_3_·3Im, despite the strong ESP maxima at an open spot near Gd atom of the second complex (Figure 2). Moreover, the intermediate complexes can transform into each other due to the small Δ*G*_535_ = +12.6 kJ/mol for the reaction GdCl_3_·3Im ⟶ GdCl_3_·2Im and Im. This value is in the range of VdW interactions. However, the energetic barrier for this reaction increases significantly at room temperature, at which Δ*G*_298_ is +46.6 kJ/mol.

The reaction in ClGdTPP·*k*Im formation may involve various numbers of imidazole molecules and produce different combinations of side products, which are hydrogen chloride (HCl) and imidazole hydrochloride (ImHCl) (Figure 4). In boiling imidazole, three initial components (H_2_TPP, GdCl_3_, Im) produce final complexes and combinations of side products by five energetically preferable processes at T = 535 K, according to negative or small positive Δ*G*_535_ which are compatible with VdW energies.

The Δ*G* dependence on temperature, rising from 298 K to 535 K through the imidazole melting point at 363 K and boiling point at 529 K, reveals linear changes for all considered reactions (Figure 4). The formation of the complex with one imidazole ligand from GdCl_3_·3Im and one free imidazole molecule (reaction V) proceeds at T = 535 K with Δ*H*_535_ = +119.8 kJ/mol and Δ*G*_535_ = +11.2 kJ/mol, which increases by up to Δ*G*_298_ = +59.8 kJ/mol at T = 298 K, which swiftly reduces the probability of ClGdTPP·Im formation at room temperature. On the other hand, the temperature decrease leads to negative Δ*G*_298_ for all ClGdTPP·2Im formations with different HCl and Im participants. At the highest temperature T = 535 K, free imidazole molecules do not participate in the exergonic but slightly endothermic reaction I with Δ*G*_535_ = −1.9 kJ/mol and Δ*H*_535_ = +18.2 kJ/mol. Two free imidazole molecules play a role in the cooling media when the next exothermic reaction, II, is the most effective among the considered processes at imidazole’s boiling point from Δ*G*_535_ = +3.4 kJ/mol through Δ*G*_530_ = 2.9 kJ/mol, and the Δ*G* = 0.0 kJ/mol at near *T* = 525 K to Δ*G*_520_ = −2.3 kJ/mol with the almost constant Δ*H* = −196.6 ± 0.2 kJ/mol. The probabilities of the other two reactions, III and IV, are also rising in the colder medium after 420 K and 470 K, respectively; these temperatures are higher than the imidazole melting point but much lower than its boiling point. Nevertheless, the highest values of Δ*G*_535_ = +9.2 kJ/mol and +16.0 kJ/mol, and of Δ*H*_535_ = −30.6 kJ/mol and −109.1 kJ/mol, are appropriate for feasibility of slightly endergonic but exothermic reactions III and IV.

On the whole, the energy dependencies on temperature demonstrate only two dominant reactions (I and II) with the lowest Δ*G* in boiling imidazole. Despite two other schemes of ClGdTPP·2Im formation reaching negative Δ*G* at room temperature, the processes cannot proceed as effectively because there are almost zero free imidazole molecules in the cold medium or chemical reaction with GdCl_3_ crystal.

### 2.3. Coordination Sphere Parameters of ClGdTPP·Im vs. ClGdTPP·2Im

**Figure 5:**
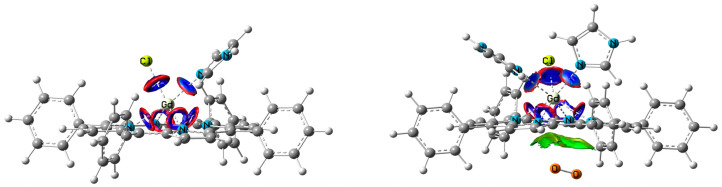
NCI representation (reduced electron density gradient isosurfaces = 0.6 a.u.) of bond types in coordinated complexes: non-covalent weak attractive coupling (dark blue), repulsive interactions or steric effects for Gd bonds (red), Van der Waals interactions with O2 only (green). The other intramolecular VdW and steric interactions are absent for clarity.

The analysis of Gd atom vicinity revealed details of bonds features that let us more precisely consider coordination parameters in two complexes (ClGdTPP·Im vs. ClGdTPP·2Im). Non-covalent interactions (NCI) [26] analysis was performed using Multiwfn 3.8 software [27,28]. The coordination number of Gd atom is defined by its weak interactions with Cl atom and imidazole ligands, as well as by four stronger bonds with N_pp_ of tetrapyrrole. In Figure 5, the thick blue and red cycles depicture the weak Gd interactions with Cl and Im moieties, whereas four thinner rings of the same colors between Gd and N_pp_ atoms concern the stronger bonds with the equatorial nitrogen centers of the tetrapyrrole. Additionally, the Van der Waals interaction with dioxygen maximally r = 3.82 Å approaching to the Gd center is depicted. The same complex without oxygen qualitatively provides the same distribution of weak interactions. The figure with the total analysis of all interactions for these structures can be found in Supplementary Materials.

Bond type can be defined by several parameters calculated at bond critical points (BCP) defined by quantum theory of atoms in molecules (QTAIM) [29] and implemented in Multiwfn. First of all, this is the Laplacian (L) of electron density, whose value is negative for covalent bonds and positive for weak interactions. Other characteristic parameters are the positive energy density H(r) for non-covalent bonds, the relation between the potential energy density V(r), and Lagrangian kinetic energy G(r) as |V(r)|/G(r), where Van der Waals interactions are indicated to be less than 1, and while covalent bonds are assumed to be more than 2. The main parameter is the bond electron density with corresponding bond length (Table 1). Interestingly, the total bond electron densities ρ are almost the same (0.34 a.u.) for 6- and 7-coordinated Gd complexes, as well as the average distance of 2.5 Å from Gd toward N_Im_, Cl and N_PP_. Approximately, bond energy is expressed as half of the potential energy density V/2. The total bond energies are −0.17 a.u. vs. for −0.16 a.u. for ClGdTPP·Im vs. ClGdTPP·2Im, which is also very close. Thus, the complexes coordinated with different numbers of imidazole ligands are characterized by almost the same averaged (bond lengths) and summed (bond electron densities, potential energy densities) parameters of coordination sphere.

### 2.4. Molecular Oxygen Attack on GdTPP Complex with Imidazole Ligands

Molecular oxygen is known as an effective triplet quencher [30] for many compounds used in medicine, but O_2_ attack could be prevented by Im ligands presenting in GdTPP complex. *T*→*T* quenching (annihilation) through energy transfer to O_2_ (sensibilization) is the most effective at position of complex of minimal distance between O_2_ and heavy atom, particularly for Gd, which responds to significant spin–orbital interaction in *S*→*T* transitions by following with phosphorescence or oxygen triplet annihilation.

**Figure 6:**
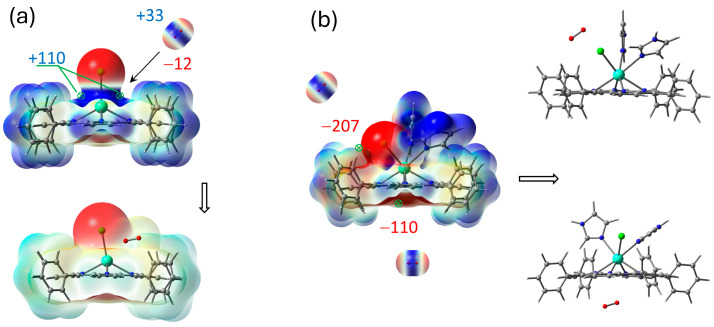
Illustration of molecular oxygen attack on ClGdTPP (**a**) and ClGdTPP·2Im, (**b**) where ESP maxima sites are in kJ/mol with scale mapping [−3 × 10^−2^ ÷ 3 × 10^−2^] a.u. ([−4 × 10^−3^ ÷ 4 × 10^−3^] a.u. for O_2_) on isodens = 0.004 ē/bohr^3^.

These interactions can be analyzed on the basis of ESP analysis. The best configuration of the complex for energy transfer from donor to oxygen acceptor is when the molecules are as close as possible with electropositive and electronegative regions (or vice versa) facing each other. Gd atom vicinity in ClGdTPP (Figure 6a) is open for molecular oxygen attack through both ESP minimum or maximum in the absence of coordination ligands. The optimized association of dioxygen and ClGdTPP complex without imidazole ligands was obtained with a distance of 2.77 Å between Gd and the attacking molecule, which could provide *T*→*T* sensibilization from the excited ClGdTPP to O_2_ with following annihilation.

Positive regions of Gd atom vicinity in ClGdTPP·2Im are shielded by coordinated Im moieties and benzene rings (Figure 6b). The most probable O_2_ attack can occur through the opened pathways toward the ESP minimum near Cl or from the opposite axial site. Two minima of −206.8 kJ/mol and −109.8 kJ/mol under the molecular plane remain to consider the interaction with O_2_ positive part. Because both Im moieties occupied the available sites to bond with Gd atom, providing a steric hindrance for the O_2_ attack on the heavy atom, the total structure consists of O_2_ at the ESP minima near Cl counterion. On the other hand, O_2_ placed on the opposite side from Gd atom cannot pass through the molecular plane to approach to the metal. Thus, imidazole ligands protect Gd atom from the oxygen attack and following potential triplet sensibilization.

The minimum distance between O_2_ and Gd in the complex with two Im moieties is 3.86 Å for the ‘top’ configuration and 3.82 Å for the ‘bottom’ oxygen (Figure 5 and Figure 6b), while |O..Gd| = 2.77 Å for ‘bare’ ClGdTPP (Figure 6a), which is crucial for excited energy transition to dioxygen. It could be added that |O–Cl| = 2.96 Å for ‘bare’ ClGdTPP vs. |O–Cl| = 3.27 Å for ‘top’ attack and closest |O–N| = 3.24 Å for ‘bottom’ attack for the complex with two imidazole ligands.

**Figure 7:**
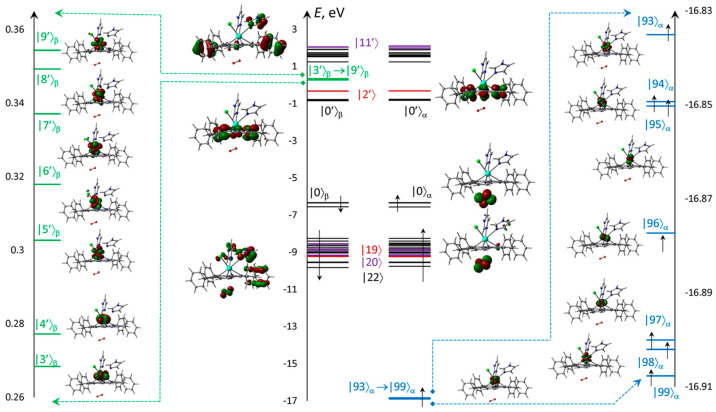
Scheme and numbering of *f*-MOs relatively to the frontier π-type |0〉 = HOMO and |0′〉 = LUMO calculated with ωB97XD&xQc for ClGdTPP·2Im·O_2_. MOs formed from *f*-AOs for vacant (green strips) and occupied (blue), as well as localized on ClGdTPP (black), on dioxygen (red), and the others with significant imidazole participation.

Electronic structure is presented for the ‘bottom’ configuration, where the vacant oxygen MOs are quite low in energy (Figure 7) to take part in long wavelength excitations and triplet–triplet transitions. The MOs energies localized mostly at the Im ligands are high enough to provide transitions only with much shorter wavelengths. The unoccupied *f*-MOs with the low energies are higher than oxygen MOs. All electronic transitions are expected from HOMO and first lower MOs. The remaining occupied MOs are significantly lower in energies, which can only create far UV short wavelength transitions. The seven deep singly occupied MOs formed almost exclusively from *f*-Aos, which provided the high multiplicity.

## 3. Computational Methods

The ωB97XD functional including both long-range correction and empirical Grimme’s D2 dispersion model from Head-Gordon and co-workers [31] was used for the optimization, as well as thermochemical and electrostatic potentials (ESP) calculations, to define the best positions for imidazole (Im) ligands (*k* = 0–2) and molecular oxygen attack of ClGdTPP·*k*Im. Functionals with long-range correction and empirical dispersion are the most suitable approaches to take into account the large size of the macrocyclic aromatic compounds centered on heavy elements [32]. The hybrid diffused polarization-consistent def2-SVP/def2-TZVP[Cl]/def2-TZVPP-ECP[Gd] (def2VP) basis sets of the Karlsruhe group [33,34] were involved to optimize and calculate properties of initial reagents (H_2_TPP, Im, GdCl_3_) as final compounds ClGdTPP·*k*Im with side products and complexes with molecular oxygen attacks. The largest relativistic all-electron hybrid ahlrichs_x2c basis sets, consisting of x2c-QZVPall-2c for Gd, x2c-TZVPall for Cl, x2c-SVP-all for H, and x2c-SVPall-2c (x2cVP) for the rest of the elements [35], which are downloaded from the Basis Set Exchange homepage [36], and were used to study electronic structures with f-MO.

Structural and thermochemical calculations were implemented in the framework of Gaussian 16 suite [37], whereas stereochemical and ESP visualization were performed in Gaussview 6 and Chemcraft 1.8 [38] graphical programs. Maxima and minima of electrostatic potentials (ESP), MO delocalization, and types of bonds were analyzed with the Multiwfn 3.8 package [27,28].

## 4. Conclusions

The detailed study of the structural parameters, thermodynamics of formation, and interaction with molecular oxygen was performed for the complexes of tetraphenylporphyrin with gadolinium chloride, which contain different numbers of coordinated imidazole molecules. ESP maxima were found to determine the number and positions of coordination bonds with imidazole molecules, two of which complete the coordination surrounding of Gd atom in ClGdTPP complexes. Thermochemical study reveals the most probable reaction pathway from initial tetraphenylporphyrin and gadolinium chloride to 7-coordinated complex. At temperature of the synthesis (535 K), the most favorable reaction involves intermediate GdCl_3_·2Im compound together with H_2_TPP and produces a final ClGdTPP·2Im complex. The analysis of coordination sphere parameters of ClGdTPP complex, including one and two imidazole ligands, revealed that these complexes are characterized by almost the same averaged bond lengths, total bond electron densities, and potential energy densities. The molecular oxygen attack on ClGdTPP·2Im complex was considered according to the ESP distribution. The minimum O_2_ approach distance of both configurations increases significantly compared to the bare complex, providing a steric hindrance of the O_2_ attack on Gd atom.

## Figures and Tables

**Table 1:** Bond lengths d in Å, bond electron densities ρ, Laplacians (L), potential energy densities V, and kinetic energies G in a.u.

	ClGdTPP·Im					ClGdTPP·2Im				
	d	ρ	L	|V|/G	V/2	d	ρ	L	|V|/G	V/2
Im						2.632	0.0350	0.1243	0.9611	−0.0144
Im	2.615	0.0365	0.1230	0.9696	−0.0153	2.631	0.0350	0.1246	0.9626	−0.0145
Cl	2.625	0.0540	0.1522	1.1843	−0.0276	2.726	0.0439	0.1234	1.1351	−0.0202
N	2.367	0.0650	0.2092	1.1285	−0.0339	2.411	0.0583	0.1909	1.0956	−0.0289
N	2.391	0.0612	0.2016	1.1068	−0.0312	2.404	0.0594	0.1942	1.1010	−0.0297
N	2.425	0.0569	0.1907	1.0852	−0.0283	2.443	0.0544	0.1815	1.0735	−0.0263
N	2.366	0.0648	0.2097	1.1269	−0.0338	2.442	0.0544	0.1818	1.0740	−0.0264
average	2.5					2.5				
total		0.34			−0.17		0.34			−0.16

## Data Availability

All published research data are available from the authors upon request.

## Supplementary Materials

The following supporting information can be downloaded at https://www.mdpi.com/article/10.3390/molecules30214246/s1, Figure S1: non-covalent weak interactions in ClGdTPP coordination complexes; Table S1: xyz coordinates of ClGdTPP·Im and ClGdTPP·2Im.
