# Supplementary material for: Computational Modelling of Imidazole Protection of Coordinated Gadolinium Tetraphenylporphyrine Against Molecular Oxygen Attack
Source: Molecules. 2025 Oct 31;30(21):4246. doi: 10.3390/molecules30214246 (PMC12608559; doi:10.3390/molecules30214246)
Supplement: Supplementary file 1 [file molecules-30-04246-s001.zip › molecules-3949191-supplementary.pdf]

Computational Modelling of Imidazole Protection of Coor-dinated Gadolinium Tetraphenylporphyrine against Mo-lecular Oxygen Attack

Vladimir Pomogaev, Daniil Lukyanov and Elena Solovyeva,*

Saint-Petersburg State University, Universitetskaya nab. 7/9, 199034 Saint Petersburg, Russia; e.solovieva@spbu.ru
* Correspondence: e.solovieva@spbu.ru

Content

1. Table S1. Coordinates of Gd coordination complexes

2. Figures S1. non-covalent weak interactions in ClGdTPP coordination complexes

ClGdTPP·2Im

Gd	-0.036220	0.389051	1.025796
Cl	-1.061924	2.177828	2.809043
N	0.037390	2.294610	-0.438463
N	2.061827	0.213075	-0.211045
N	-2.072404	0.269137	-0.259936
N	-0.053356	-1.818403	-0.021306
C	-1.045868	3.103754	-0.573837
C	-0.611967	4.461337	-0.840183
C	0.745623	4.442748	-0.855130
C	1.147006	3.073469	-0.592124
C	2.476856	2.624216	-0.542730
C	2.888481	1.280027	-0.400799
C	4.260091	0.825397	-0.468760
C	4.231285	-0.531818	-0.341783
C	2.841893	-0.906635	-0.188499
C	2.380131	-2.229167	-0.037865
C	1.034762	-2.640919	-0.013444
C	0.602102	-4.022096	-0.082802
C	-0.756032	-4.001129	-0.160662
C	-1.155797	-2.609508	-0.117779
C	-2.491324	-2.157597	-0.181345
C	-2.901174	-0.814185	-0.239168
C	-4.280376	-0.367715	-0.297218
C	-4.252049	0.989508	-0.367738
C	-2.855982	1.379577	-0.364352
C	-2.393021	2.702408	-0.485310
H	1.421567	5.273115	-1.045361
H	5.131035	1.460024	-0.615149
H	5.074770	-1.218173	-0.364506
H	1.258138	-4.889222	-0.113950
H	-1.428822	-4.849240	-0.264550
H	-5.155294	-1.013558	-0.289286
H	-5.096952	1.671385	-0.429242
C	3.548298	3.657758	-0.681492
C	4.281483	3.779520	-1.867714
C	3.827954	4.527696	0.379063
C	5.276681	4.747744	-1.989632
H	4.060785	3.108152	-2.700956
C	4.822759	5.496441	0.257373
H	3.252496	4.440347	1.303956
C	5.550465	5.608101	-0.926844
H	5.837974	4.833738	-2.922767
H	5.029632	6.168669	1.093117
H	6.329001	6.368134	-1.023045
C	3.414375	-3.303987	0.068005
C	4.109605	-3.757094	-1.058710
C	3.697696	-3.877732	1.313054
C	5.069848	-4.760843	-0.942269

H	3.887606	-3.315803	-2.033138
C	4.657578	-4.881654	1.430722
H	3.153967	-3.527550	2.194447
C	5.346685	-5.325287	0.302571
H	5.602554	-5.107008	-1.830807
H	4.867927	-5.320709	2.408670
H	6.098060	-6.112814	0.392873
C	-3.563004	-3.199025	-0.205409
C	-3.840478	-3.958214	0.937760
C	-4.308460	-3.433401	-1.367091
C	-4.842860	-4.926391	0.921348
H	-3.256372	-3.779882	1.843494
C	-5.310534	-4.401998	-1.384628
H	-4.092969	-2.847809	-2.263822
C	-5.581515	-5.150738	-0.239900
H	-5.049160	-5.509791	1.821802
H	-5.880964	-4.575054	-2.299925
H	-6.366684	-5.909903	-0.253707
H	-1.269839	5.309851	-1.012823
C	-3.418404	3.789466	-0.497556
C	-4.179765	4.068032	-1.637272
C	-3.617740	4.547827	0.662523
C	-5.127427	5.090828	-1.619432
H	-4.021079	3.478023	-2.543244
C	-4.566376	5.568745	0.678478
H	-3.019495	4.319567	1.549022
C	-5.322455	5.842800	-0.461414
H	-5.714082	5.303116	-2.516183
H	-4.715925	6.154006	1.588617
H	-6.064307	6.644583	-0.447815
N	1.681290	-0.020614	2.976064
C	2.996153	-0.075871	2.951260
N	3.496893	-0.107122	4.204590
C	2.439075	-0.062911	5.084032
C	1.319187	-0.006229	4.301779
H	3.620577	-0.090847	2.059674
H	4.478167	-0.142700	4.444358
H	2.574092	-0.063559	6.161087
H	0.276493	0.075681	4.596766
N	-1.300805	-1.175375	2.722952
C	-2.427401	-0.852680	3.316955
N	-2.865507	-1.870344	4.090234
C	-1.961024	-2.903250	3.983456
C	-0.997296	-2.451107	3.122877
H	-2.925050	0.111768	3.223009
H	-3.709649	-1.865084	4.646347
H	-2.081338	-3.843193	4.513303
H	-0.113942	-2.968653	2.753667

ClGdTPP·Im

Gd	-0.255661	-0.059492	0.919065
Cl	-1.050797	1.145402	3.111660
N	-0.118604	1.999708	-0.239232
N	1.904203	-0.077449	-0.106949
N	-2.234886	-0.060111	-0.377319
N	-0.207113	-2.147529	-0.312323
C	-1.210422	2.807770	-0.353843
C	-0.777426	4.187805	-0.453466
C	0.579592	4.181687	-0.390523
C	0.987237	2.797863	-0.244664
C	2.318461	2.353399	-0.149910
C	2.733447	1.004635	-0.107887
C	4.110073	0.556505	-0.103139
C	4.081536	-0.803464	-0.127920
C	2.687452	-1.196935	-0.137822
C	2.232344	-2.525688	-0.234991
C	0.892344	-2.951490	-0.369465
C	0.472074	-4.325008	-0.550211
C	-0.889891	-4.317080	-0.614681
C	-1.305702	-2.939763	-0.465992
C	-2.643823	-2.488504	-0.509549
C	-3.060029	-1.146124	-0.482430
C	-4.432491	-0.694341	-0.581256
C	-4.405547	0.664850	-0.544414
C	-3.016209	1.056634	-0.425506
C	-2.553769	2.388203	-0.395198
H	1.252964	5.033242	-0.453200
H	4.984713	1.202537	-0.102565
H	4.930107	-1.482693	-0.161106
H	1.132061	-5.185051	-0.635304
H	-1.553873	-5.164181	-0.772462
H	-5.303435	-1.339411	-0.670843
H	-5.249355	1.348523	-0.598774
C	3.383435	3.401991	-0.113584
C	4.202036	3.632760	-1.225386
C	3.570881	4.175503	1.038019
C	5.190108	4.614952	-1.185397
H	4.054755	3.036094	-2.128745
C	4.559079	5.157577	1.078205
H	2.931347	4.001208	1.906774
C	5.371456	5.379161	-0.033153
H	5.819199	4.786927	-2.061595
H	4.695057	5.752460	1.984054

H	6.145269	6.149325	-0.002142
C	3.275904	-3.593565	-0.164164
C	3.639849	-4.339096	-1.290822
C	3.907240	-3.860689	1.057464
C	4.609307	-5.336534	-1.196963
H	3.157554	-4.127977	-2.248074
C	4.876914	-4.857178	1.151154
H	3.634243	-3.269140	1.935816
C	5.228915	-5.599568	0.024184
H	4.886061	-5.908344	-2.085458
H	5.363771	-5.053215	2.109346
H	5.989868	-6.379668	0.096340
C	-3.705957	-3.534229	-0.630140
C	-4.070559	-4.306340	0.478703
C	-4.347579	-3.761262	-1.853136
C	-5.057009	-5.284798	0.368332
H	-3.577077	-4.128863	1.437385
C	-5.333596	-4.740006	-1.964624
H	-4.064542	-3.162237	-2.721844
C	-5.690627	-5.504164	-0.854191
H	-5.335269	-5.876088	1.243520
H	-5.824618	-4.907662	-2.925745
H	-6.464124	-6.270250	-0.941400
H	-1.435224	5.044970	-0.576481
C	-3.590996	3.465744	-0.403434
C	-4.231481	3.845950	-1.587424
C	-3.927182	4.109021	0.793236
C	-5.193480	4.854995	-1.576180
H	-3.967582	3.345405	-2.522186
C	-4.889930	5.116836	0.803252
H	-3.426441	3.807145	1.716678
C	-5.524432	5.492076	-0.380717
H	-5.685346	5.146289	-2.506966
H	-5.146455	5.610887	1.742953
H	-6.278107	6.282627	-0.371900
N	0.826514	-1.698378	2.645889
C	0.653505	-2.996378	2.764361
N	1.429937	-3.493282	3.750691
C	2.145295	-2.450069	4.291628
C	1.751674	-1.340702	3.593403
H	-0.003548	-3.611447	2.150976
H	1.482726	-4.464759	4.025744
H	2.843491	-2.582856	5.112093
H	2.062199	-0.306742	3.724415

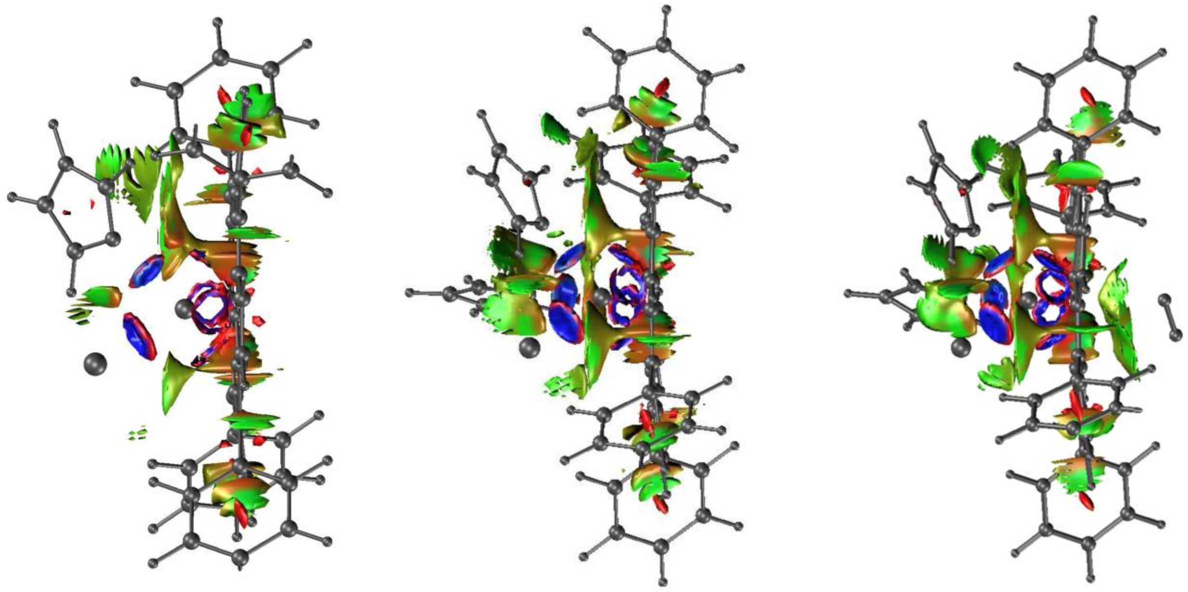

Figure S1. NCI representation (reduced electron density gradient isosurfaces = 0.6 a.u.) of bond types in co-ordinated complexes: non-covalent weak attractive coupling (dark blue), Van-der-Waals interac-tions (green) and repulsive interactions or steric effects (red).
